# Genetic mapping of the female mimic morph locus in the ruff

**DOI:** 10.1186/1471-2156-14-109

**Published:** 2013-11-20

**Authors:** Lindsay L Farrell, Terry Burke, Jon Slate, Susan B McRae, David B Lank

**Affiliations:** 1 Department of Animal and Plant Sciences, University of Sheffield, Sheffield, S10 2TN, UK; 2 Department of Biological Sciences, Simon Fraser University, Burnaby, BC V5A 1S6, Canada; 3 Department of Biology and Center for Biodiversity, East Carolina University, Greenville, NC 27858-4353, USA

## Abstract

**Background:**

Ruffs (Aves: *Philomachus pugnax*) possess a genetic polymorphism for male mating behaviour resulting in three permanent alternative male reproductive morphs: (i) territorial ‘Independents’, (ii) non-territorial ‘Satellites’, and (iii) female-mimicking ‘Faeders’. Development into independent or satellite morphs has previously been shown to be due to a single-locus, two-allele autosomal Mendelian mode of inheritance at the *Satellite* locus. Here, we use linkage analysis to map the chromosomal location of the *Faeder* locus, which controls development into the Faeder morph, and draw further conclusions about candidate genes, assuming shared synteny with other birds.

**Results:**

Segregation data on the *Faeder* locus were obtained from captive-bred pedigrees comprising 64 multi-generation families (*N* = 381). There was no evidence that the *Faeder* locus was linked to the *Satellite* locus, but it was linked with microsatellite marker *Ppu020*. Comparative mapping of ruff microsatellite markers against the chicken (*Gallus gallus*) and zebra finch (*Taeniopygia guttata*) genomes places the *Ppu020* and *Faeder* loci on a region of chromosome 11 that includes the *Melanocortin-1 receptor* (*MC1R*) gene, which regulates colour polymorphisms in numerous birds and other vertebrates. Melanin-based colouration varies with life-history strategies in ruffs and other species, thus the *MC1R* gene is a strong candidate to play a role in alternative male morph determination.

**Conclusion:**

Two unlinked loci appear to control behavioural development in ruffs. The *Faeder* locus is linked to *Ppu020*, which, assuming synteny, is located on avian chromosome 11. *MC1R* is a candidate gene involved in alternative male morph determination in ruffs.

## Background

**Figure 1:**
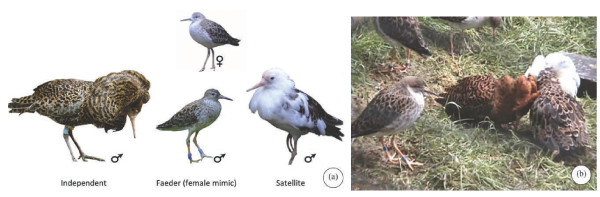
**(a) Three permanent alternative male reproductive morphs of ruff (*Philomachus pugnax*) associated with territorial lekking behaviour and plumage colour.** Pictured bottom left: territorial dark-plumed ‘Independent’; bottom middle: female mimic ‘Faeder’; bottom right: non-territorial white-plumed ‘Satellite’; top middle: female (photos by L.L.F and S.B.M). **(b)** On a captive lek, the independent and satellite males are displaying (right) with the faeder male near by (left) (photo by S.B.M).

Evolving and maintaining genetic polymorphisms responsible for large phenotypic differences remains a subject of interest, despite >70 years of study (e.g. [1,2]). Genomic methods now enable polymorphisms to be described down to the genetic and molecular expression levels (e.g. [3]). Ruffs (*Philomachus pugnax*) possess three distinct permanent alternative male reproductive morphs that differ in territorial lekking behaviour, plumage colour, and size: dark-plumed territorial ‘Independents’, white-plumed non-territorial ‘Satellites’ and small female mimic ‘Faeders’ that lack display plumage and behaviour [4-7]. Development into independent or satellite morphs has been previously shown to be due to a genetic polymorphism consistent with a single-locus, two-allele autosomal Mendelian mode of inheritance at the *Satellite* locus, with a dominant *S* allele producing satellites [8,9]. Genetically, independent males are homozygous recessive at the *Satellite* locus and *ca* 90% of satellites should be heterozygotes [8]. Remarkably, only as recently as 2006, a third morph was discovered: faeder males resemble large females, completely lacking any ornamental breeding plumage during the breeding season [5] (Figure 1). It was recently reported that a dominant allele controls development into both faeders and diminutive females, coined ‘faeder females’ [10]. Whether the faeder allele is at the same *Satellite* locus, or a separate locus, has yet to be determined, as more detailed pedigree-based genetics of the newly discovered morph are not yet available.

Prior to each breeding season, independent and satellite males grow ornamental plumage that includes a feather ‘ruff’ and ‘head tufts’, which are each individually distinctive in colour and pattern and fixed for life [11,12]. At leks, independents establish and defend small breeding courts where they perform a variety of territorial threat displays and fight against other independents. The white-plumed satellites do not hold territories, are rarely aggressive, and are actively courted into co-displaying on courts held by independents, apparently due to female preference for male-male cooperation on leks [4,6,7,13,14] and a high rate of polyandry [15]. In contrast to both classes of ornamented males, faeder males grow breeding plumage that is similar to that of females–lacking display feathers–and aggregate close to displaying males to ‘sneak’ copulations with females and interfere with copulation attempts by other males ( [5]; Lank *et al*. unpublished) (Figure 1). Females believed to be carrying the dominant *Faeder* allele form a distinct small size mode [10]. Normal-sized females carrying the dominant *Satellite* allele can be identified from the phenotype ratios of their male offspring when mated to independent males, and/or confirmed with observations of behaviour and ornamental plumage growth when implanted with testosterone [9].

Recently, a microsatellite linkage map for the ruff was constructed, identifying seven linkage groups and a further five single-marker loci homologous to locations on known chicken (*Gallus gallus*) and zebra finch (*Taeniopygia guttata*) chromosomes [16]. As a step towards identifying the genes underlying the morph polymorphisms, we attempted to map the causal satellite and faeder loci by using linkage analysis to identify markers that co-segregated with each morph type in a pedigreed and phenotyped breeding population.

## Methods

Pedigree, phenotype, and microsatellite information were available from 381 individuals from a captive population of ruffs spanning fourteen breeding years and comprising 64 families (*N* = 381 individuals, [10,16]). In total, 167 individuals were included for the *Satellite* locus: 129 assigned as independents (120 males, 9 females), 38 satellites (35 males, 3 females) and 381 individuals for the *Faeder* locus: 43 faeders (24 males, 19 females) and 338 non-faeders (155 males, 183 females).

This research was conducted at Simon Fraser University under approval of the Animal Care Committee.

### Linkage analysis

Separate autosomal genetic models for the two male behavioural polymorphisms (Satellite *versus* Independent; Faeder *versus* Not Faeder) were tested in CRIMAP v.2.4 [17] using phenotypic and pedigree data to assign putative genotypes separately for both the *Satellite* and *Faeder* loci. For the *Satellite* locus: independent males (*N* = 120) were coded as homozygous recessive (*ss*) and satellite males (*N* = 35) coded with the dominant *S* allele (*S*_), with faeders not coded at this locus. A small number of females (*N* = 12) were assigned a satellite or independent behavioural morph and putative genotype based on pedigree analysis of their male offspring morph ratios when mated with an independent male (Lank *et al.* unpublished). Females mated with an independent male that produced mixed offspring were designated as heterozygotes (*Ss*, *N* = 3), and females with a high number of offspring (*N* = 11–22) who failed to produce any satellites when mated with independents were designated as homozygous recessive at the *Satellite* locus (*ss*, N = 9). In the majority of cases, these morph assignments were confirmed with testosterone-induced behavioural data [9]. For the *Faeder* locus: both independent and satellite males were coded as homozygous recessive (*ff*, *N* = 155) and faeder males as (*F_*), indicating that they carry at least one copy of the *F* allele (*N* = 24) [10]. Since the faeder frequency in natural populations is *ca* 1% [5,18-20], the probability of observing homozygous faeders in the wild is low. Faeders in the captive population were derived from 2 wild-caught founders. Both of these males produced both faeder and non-faeder offspring when mated exclusively with females from non-faeder lineages, as did their sons. No faeder daughters are included as mothers in these analyses. For females, phenotypic assignments as ‘faeder females’ (*N* = 19) were made through principal component analysis of size distributions based on tarsus, culmen, and minimum mass [10]. All non-faeder females (*N* = 183) were coded as homozygous recessive (*ff*), and faeder females coded as (*F_*) for similar reasons as were the males.

A test for linkage between the *Satellite* locus and *Faeder* locus, and all microsatellite markers (*N* = 58) used in the ruff microsatellite linkage map [16], was performed by means of the two-point function in CRIMAP, with a LOD score >3.0 being taken as evidence of linkage. The *Satellite* and *Faeder* loci were first run separately, then together in CRIMAP. We used comparative mapping [21,22] of microsatellite markers used in the ruff microsatellite linkage map [16] against the chicken and zebra finch genome assemblies to search for possible candidate genes in the genomic location close to any microsatellites that were linked to the ruff *Faeder* locus.

## Results and discussion

No linkage was detected between the *Satellite* and *Faeder* loci, and the *Satellite* locus was unlinked to any other marker in twopoint analysis. The latter result may be due in part to the low number of satellites with heterozygous genotypes and high number of independents contained within the pedigree, resulting in a small number of informative meioses at the target *Satellite* locus. Out of the total 167 individuals with inferred genotypes at the *Satellite* locus, 129 of these were independents and 38 were satellites. The non-linkage of the two behavioural loci, *Satellite* and *Faeder*, to the same marker or, more importantly, to each other, indicates that two independent loci determine alternative morph development in ruffs. Additional genotyping of satellite individuals and/or more detailed pedigree data will further test this two-locus model.

Several species with three heritable alternative mating phenotypes have been described (e.g., [23]), but explicit mendelian models have been best tested for the marine isopod *Paracerceis sculpta*[24], for which a 1-locus 3-allele model with hierarchical dominance was supported. Remarkably, alleles coding for ‘alternative’ morphs in these other systems are dominant to those of the presumed ancestral allele, as they are in the ruff [10]. In the ruff, this suggests a sequence for invasion by these derived morphs, with faeders following satellites.

In the twopoint analyses, the *Faeder* locus was strongly linked to microsatellite marker *Ppu020* with a LOD score 8.24 and recombination fraction of 0.03*.* This locus was not placed on the ruff linkage map but comparative mapping has shown it to be on chromosome 11 [16]. Further linkage analysis with microsatellite markers on chromosome 11 was not possible, however, due to the small number of markers genotyped on this chromosome in the ruff linkage map [16].

**Figure 2:**
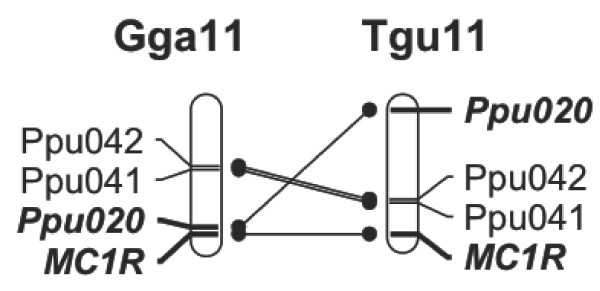
**In chicken, the distance between the *Melanocortin-1 receptor *(MC1R) gene and *Ppu020 *is 1.2 Mb, and in zebra finch, the distance is 20.7 Mb.** An interchromosomal rearrangement between ruff microsatellite markers *Ppu020/Faeder* and Ppu042/Ppu041 is visible between the homologues of chicken and zebra finch.

By comparative mapping [21,22] of ruff microsatellite markers [25] to the chicken and zebra finch genome assemblies (Figure 2), an obvious candidate locus was identified. The *Melanocortin-1 receptor* (*MC1R*) gene, an important pigment-regulating gene in birds and numerous other vertebrates, is located on chromosome 11 in both species. In chicken, the distance between *Ppu020* and *MC1R* is 1.2 Mb and, in zebra finch, the distance is 20.7 Mb (Figure 2).

Although ruff microsatellite *Ppu020* is not included in the ruff linkage map for chromosome 11, two further ruff microsatellite loci have been assigned to this chromosome by *in silico* comparative mapping [25]. Comparison of the locations of these markers in the zebra finch and chicken genomes indicates that there was an intrachromosomal rearrangement of this region of chromosome 11 in an unknown lineage since the divergence of the ancestors of chicken and zebra finch (Figure 2). Therefore, inferring the physical distance between *MC1R* and the *Faeder* locus in ruffs is not straightforward, especially as no species in the ruff’s avian superorder (the Charadriiformes) has yet been the subject of a full genome sequencing project.

## Conclusion

Regardless of the precise location of *MC1R* in ruffs, we conclude that this gene and those in proximity to it are candidates for the *Faeder* locus. Melanin-based colouration has previously been shown to be associated with morphology, physiology, life-history strategies and behaviour in several bird species (e.g., [26-28]), including ruffs, as well as having correlated fitness-related effects in other vertebrates [29,30].

## Competing interests

The authors declare no competing interests.

## Authors’ contributions

LLF designed the study, performed the laboratory work, completed the data analysis, and drafted and revised the manuscript. DBL, TB and JS helped with the design of the study, the interpretation of the data, and with drafting and revision of the manuscript. JS and TB conceived coding the faeder behaviour locus into the linkage map. SBM contributed to the development of the ruff pedigree and quantifying faeder phenotypes with DBL. All authors read and approved the final manuscript.
